# Increased longevity mediated by yeast NDI1 expression in *Drosophila* intestinal stem and progenitor cells

**DOI:** 10.18632/aging.100595

**Published:** 2013-09-06

**Authors:** Jae H. Hur, Sepehr Bahadorani, Jacqueline Graniel, Christopher L. Koehler, Matthew Ulgherait, Michael Rera, D. Leanne Jones, David W. Walker

**Affiliations:** ^1^ Department of Integrative Biology and Physiology, University of California, Los Angeles, Los Angeles, CA 90095, USA; ^2^ Laboratory of Genetics, The Salk Institute for Biological Studies, La Jolla, CA 92037, USA; ^3^ Department of Molecular, Cell and Developmental Biology, University of California, Los Angeles, Los Angeles, CA 90095, USA; ^4^ Molecular Biology Institute, University of California, Los Angeles, Los Angeles, CA 90095, USA

**Keywords:** dilp2, lifespan, intestinal barrier, insulin/insulin-like growth factor signaling, mitochondria

## Abstract

A functional decline in tissue stem cells and mitochondrial dysfunction have each been linked to aging and multiple aging-associated pathologies. However, the interplay between energy homeostasis, stem cells, and organismal aging remains poorly understood. Here, we report that expression of the single-subunit yeast alternative NADH dehydrogenase, *ndi1*, in *Drosophila* intestinal stem and progenitor cells delays the onset of multiple markers of intestinal aging and extends lifespan. In addition, expression of ndi1 in the intestine increases feeding behavior and results in organismal weight gain. Consistent with increased nutrient uptake, flies expressing *ndi1* in the digestive tract display a systemic reduction in the activity of AMP-activated protein kinase (AMPK), a key cellular energy sensor. Together, these results demonstrate that *ndi1* expression in the intestinal epithelium is an effective strategy to delay tissue and organismal aging.

## INTRODUCTION

Identifying the molecular and cellular mechanisms that underlie organismal aging represents an urgent biomedical challenge. Towards this goal, considerable attention has been focused on the progressive decline in stem cell functions [1] and, separately, mitochondrial activity [2] that occurs during aging. Fundamental questions remain, however, regarding the relationships among mitochondrial activity within stem cell populations, tissue homeostasis, and organismal aging. Nutrient intake is closely related to energy homeostasis, stem cell maintenance and lifespan determination [3]. Indeed, moderate dietary restriction (DR) can delay the onset of pathology and extend lifespan in diverse species, from yeast to primates [4]. Similarly, many of the genetic mutations that have been reported to extend organismal lifespan are thought to decrease the activity of nutrient signaling pathways, such as the insulin/ insulin-like growth factor signaling (IIS), and the target of rapamycin (TOR) signaling pathways [5]. Critically, the specifics of how alterations in tissue or organ homeostasis affects nutrient signaling pathways and aging of the whole organism remain poorly understood.

The integrity of the intestinal epithelium is essential for maintaining barrier function, nutrient uptake, metabolic homeostasis, and hence, organismal health and survival. In *Drosophila*, the midgut epithelium is maintained by multipotent intestinal stem cells (ISCs), which are distributed along the basement membrane [6, 7]. Division of an ISC gives rise to one daughter cell that retains stem cell fate and another daughter cell that becomes an enteroblast (EB). During aging, there is a dramatic increase in ISC proliferation which is accompanied by the accumulation of cells that express markers of both ISCs and terminally differentiated daughter cells [8, 9]. In addition, loss of intestinal barrier function has been shown to accompany aging across a range of *Drosophila* genotypes and environmental conditions [10]. Moreover, the age-dependent loss of intestinal integrity is linked to multiple markers of organismal aging, including systemic metabolic dysfunction, increased expression of immunity-related genes, reduced spontaneous physical activity and, critically, is a harbinger of death [10]. Recently, we have characterized the role of the *Drosophila* PGC-1 homolog (*dPGC-1*/*spargel*), a key regulator of mitochondrial energy metabolism, in the maintenance of ISC quiescence, intestinal integrity, and lifespan determination [11]. More specifically, up-regulation of *dPGC-1* in ISC/EBs delays the onset of markers of intestinal aging and confers increased longevity. However, given the diverse roles that PGC-1 plays in metabolism [12], the question of whether an increase in mitochondrial activity alone, in ISC lineages, is sufficient to confer these phenotypic outcomes remains to be determined.

The single subunit alternative internal NADH dehydrogenase (*ndi1*) from *Saccharomyces cerevisiae*, which lacks a conventional electron transport chain (ETC) complex I, can function in *Drosophila* mitochondria and is able to complement and supplement endogenous ETC complex I [13-15]. Here, we expressed *ndi1* in *Drosophila* somatic stem cell lineages and examined its impact on tissue and organismal aging. *ndi1* expression in ISCs/EBs improves tissue homeostasis in the aging intestine and confers increased longevity at the organismal level, demonstrating that increased NADH dehydrogenase activity alone is sufficient to produce these beneficial effects. Among other phenotypes associated with increased longevity, we find that flies with ISC/EB-specific *ndi1* expression display increased feeding behavior and whole body alterations in metabolic signaling pathways. Consistent with an increase in nutrient intake, long-lived *ndi1* flies show a systemic reduction in the activity of AMP-activated protein kinase (AMPK), a key cellular energy sensor [16]. Our results reveal novel roles for a NADH dehydrogenase in modulating stem cell behavior and intestinal homeostasis during aging. Moreover, we show that enhanced mitochondrial complex I activity in ISC lineages can simultaneously alter feeding behavior in adult flies and prolong lifespan.

## RESULTS

### Expression of *ndi1* in intestinal stem and progenitor cells extends lifespan

**Figure 1:**
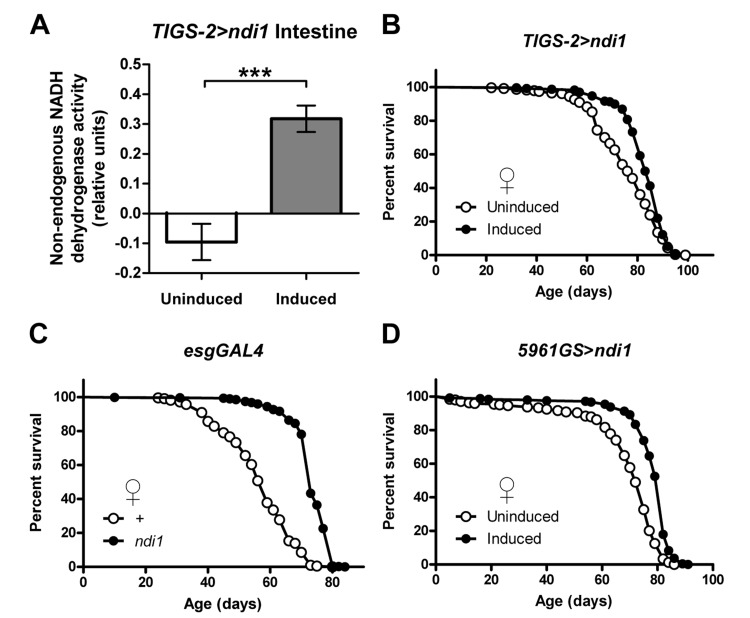
Intestine-specific expression of *ndi1* increases lifespan (**A**) Analysis of NDI1 enzymatic activity in mitochondria isolated from intestines. NDI1 is expressed by transgenic expression of an *ndi1* cDNA under control of the intestine-specific *TIGS-2* driver (*TIGS-2>ndi1*). Transgenic expression is induced by exposure of flies to the drug RU486 (100mg/l). Expression of *ndi1* is sufficient to confer flavone sensitive, rotenone insensitive NADH dehydrogenase activity to mitochondria isolated from intestines. (***p<0.001, t test, 5 replicates per condition, mitochondria from 10 dissected intestines from female flies per replicate). (**B**) Survival curves of female *TIGS-2*>*ndi1* flies with or without RU486-mediated transgene induction. Constitutive expression of *ndi1* by RU486 exposure (10mg/l during development, 50mg/l during adulthood) increases lifespan (p<0.0001, log-rank test, at least 200 flies per condition). (**C**) Survival curves of female *esgGAL4*>*ndi1* flies compared to isogenic controls. *UAS-ndi1* and the isogenic control strain (*w^1118^*) were crossed to *esgGAL4*. A 50% increase in mean survival was observed in response to *ndi1* expression (p<0.0001, log-rank test, at least 200 flies per condition). (**D**) Survival curves of female *5961GS*>*ndi1* flies with or without RU486-mediated transgene induction. Adult-onset expression of *ndi1* by RU486 exposure (0.5mg/l) increases fly lifespan (p<0.0001, log-rank test, at least 200 flies per condition).

The intestine is a critical target organ with respect to genetic manipulations that can extend longevity [17], as has been shown previously with *dPGC-1* upregulation [11]. To better understand the relationships among mitochondrial respiratory chain activity, intestinal homeostasis, and lifespan determination, we expressed a previously described *UAS-ndi1* construct [14, 15] in the *Drosophila* intestine using the intestine-specific RU486-inducible Gene-Switch driver line *TIGS-2* [18]. Unlike the endogenous *Drosophila* ETC complex I which is sensitive to rotenone inhibition but insensitive to flavone, NDI1 is insensitive to rotenone but inhibited by flavone [19]. Induced expression of *ndi1* in the adult intestine produced a robust rotenone-insensitive, flavone-sensitive NADH dehydrogenase activity in mitochondria isolated from intestines (Figure 1A). Control flies from the same background strain that were not provided RU486 did not show detectable levels of rotenone-insensitive, flavone-sensitive NADH dehydrogenase activity, supporting the fidelity of the Gene-Switch system [20, 21] and functionality of the *ndi1* transgene and NDI1 protein in the adult fly intestine.

We used this system to examine the impact of intestine-specific expression of *ndi1* on *Drosophila* lifespan. Induced expression of *ndi1* using the *TIGS-2* driver throughout the life of the fly resulted in a significant increase in lifespan in female flies (Figures 1B and S1A) and no major effect in male flies. RU486 produced no major effects on longevity in control flies (Figure S1B). To examine the impact of targeted expression of *ndi1* in intestinal stem cell lineages (ISCs and EBs), we first used the constitutive *esgGAL4* driver line and observed a significant extension of lifespan in both female (Figures 1C and S1C-D) and male flies (Figures S1E-F) compared to controls. *esgGal4* expression is restricted to ISCs and EBs in the intestine, however, it is also expressed in stem cells within malpighian tubules, germline and somatic stem cells in the testis, and in salivary glands [22]. Therefore, to validate and extend this finding we took advantage of the RU486-inducible *5961GS* driver which recapitulates the *esgGal4* expression pattern in the digestive tract (ISCs/EBs and malpighian tubule stem cells) [22, 23] but is not expressed in salivary glands [22] or testis (C.L.K. and D.L.J., unpublished data). Induced expression of *ndi1* during adulthood, via *5961GS*, resulted in a significant lifespan increase in females (Figures 1D and S1G-I) but not in males (Figures S1J-S1K). Expression of *ndi1* during adulthood using a Gene-Switch driver that is expressed in EBs and post mitotic enterocytes (ECs) (*5966GS*, [23]) failed to increase lifespan (Figures S1L-M), implicating expression in ISCs as the major contributor to longevity. The largest and most consistent lifespan extension phenotypes using *ndi1* expression were observed with female flies. Therefore, unless noted otherwise, we focused our studies to female flies for the remainder of this study.

### *ndi1* expression in ISCs/EBs improves markers of intestinal homeostasis during aging

**Figure 2:**
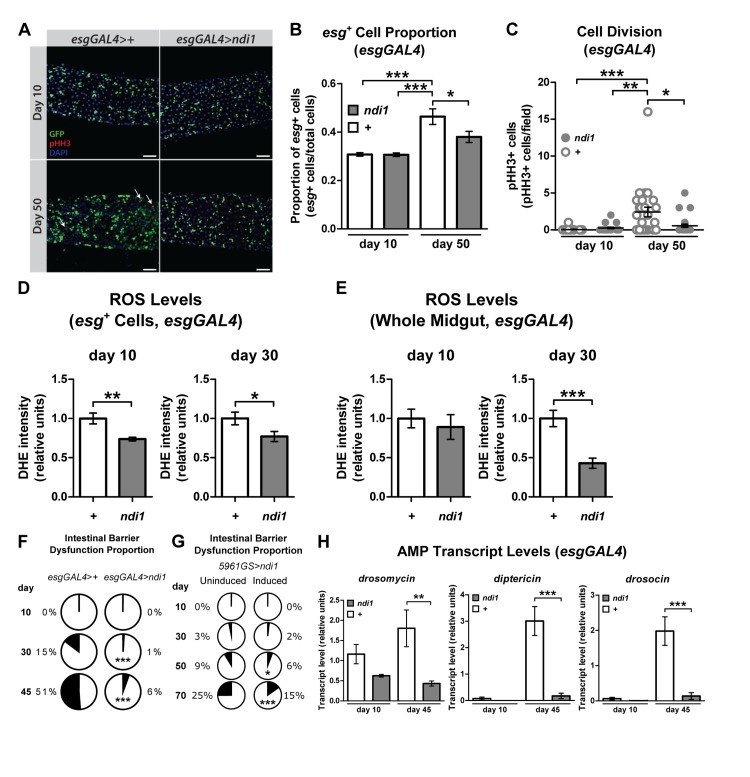
*ndi1* expression maintains intestinal homeostasis during aging (**A**) Immunofluorescence images evaluating intestinal homeostasis during aging. Control flies (*esgGAL4*>+, upper panel) and *ndi1* expressing flies (*esgGAL4*>*ndi1*, lower panel) were assayed for *esg^+^* cells (GFP^+^ cells) and mitotic cells (pHH3^+^ cells, arrows) 10 days and 50 days post eclosion. Scale bars=50μm. (**B**) Quantification of proportion of *esg*^+^ cells. The proportion of *esg*^+^ cells (GFP^+^ cells) in all cells (DAPI stain) was increased in aged control flies (“*+*”, *esgGAL4>+*), but not in *ndi1* expressing flies (“*ndi1*”, *esgGAL4>ndi1*). (*p<0.05, ***p<0.001, One-way ANOVA with Tukey's post hoc test, at least 22 flies per condition). (**C**) Quantification of mitotic cells per field of view. The median number of mitotic events (pHH3^+^ cells per field of view) is elevated in aged control flies (“*+*”, *esgGAL4*>+), but not in *ndi1* expressing flies (“*ndi1*”, *esgGAL4>ndi1*). (*p<0.05, **p<0.01, ***p<0.001, Kruskal-Wallis test followed by Dunn's multiple comparisons, at least 22 flies per condition). (**D**)Quantification of ROS levels per area in ISCs/EBs. *ndi1* expression (“*ndi1*”, *esgGAL4>ndi1*) decreases DHE fluorescence within *esg^+^* cells(GFP^+^ cells, arrows in figure S2A) in both 10 and 30 day old intestines compared to isogenic controls (“*+*”, *esgGAL4>+*).(*p<0.05, **p<0.01, t test, at least 3 images per gut, 10 guts per condition). (**E**) Quantification of ROS levels per area in midguts. *ndi1* expression in ISCs/EBs (“*ndi1*”, *esgGAL4>ndi1*) results in decreased DHE fluorescence in gut tissues relative to isogenic controls (“*+*”, *esgGAL4>+*) at 30 days post eclosion. (***p<0.001, t test, at least 3 images per gut, 10 guts per condition). (**F**) Proportion of flies showing loss of intestinal integrity as a function of age, assayed using blue dye no. 1. Aged flies that express *ndi1* in ISCs/EBs (*esgGAL4>ndi1*) show reduced levels of intestinal barrier dysfunctionrelative to controls (*esgGAL4>+*). (***p<0.001, binomial test, at least 190 flies per condition). (**G**) Proportion of flies showing loss of intestinal integrity as a function of age in *5961GS*>*ndi1* flies with or without RU486-mediated transgene induction. Adult-onset expression of *ndi1* by RU486 exposure (0.5mg/l) improves maintenance of intestinal integrity during aging. (**H**) Systemic expression of *Drosomycin, Drosocin* and *Diptericin* in 10 and 45 day old flies. Aged flies that express *ndi1* in ISCs/EBs (“*ndi1*”, *esgGAL4>ndi1*) show reduced expression of antimicrobial peptides (AMPs) relative to controls (“*+*”, *esgGAL4>+*). (**p<0.01, ***p<0.001, t test, 5 replicates per condition, 5 flies per replicate).

Homeostasis of the digestive tract has been shown to play a central role in lifespan determination in *Drosophila* [10, 11, 17, 22]. Therefore, we examined markers of intestinal homeostasis in flies that express *ndi1* in ISCs/EBs. First, we set out to determine whether *ndi1* could delay the onset of markers of ISC proliferation and the accumulation of misdifferentiated ISC daughter cells reported to occur in the aged midgut [8, 9]. Consistent with improved intestinal tissue homeostasis, examination of aged flies that express *ndi1* in ISCs/EBs along with an *esg* reporter (*UAS-gfp*) revealed a significant decrease in the number of *esg* positive cells in the midgut relative to controls (Figure 2A-B). In addition, we also observed a delay in the precocious activation of ISC proliferation, as measured by phosphorylation of histone H3 (pHH3), a marker of cell cycle progression through mitosis. Female flies, 50 days post eclosion, expressing *ndi1* under the control of *esgGAL4* driver contained significantly fewer pHH3^+^ cells, when compared to controls (Figure 2C). No difference in the number of pHH3^+^ cells was observed in 10 day old flies, indicating that *ndi1* expression specifically delays the age-related increase in ISC proliferation.

An increase in reactive oxygen species (ROS) has been implicated in the loss of tissue homeostasis in the aged fly intestine [24, 25]. Previously, we reported that pan-neuronal expression of *ndi1* can reduce ROS levels in the aged brain [15]. It is unclear, however, whether *ndi1* expression only in progenitor cells of a tissue is sufficient to cause such changes throughout the tissue. To test this idea, we examined the endogenous levels of ROS in the intestines of control and *esgGAL4>ndi1* flies using dihydroethidium (DHE), a redox-sensitive dye that exhibits increased fluorescence intensity when oxidized [26]. Targeted expression of *ndi1* in ISCs/EBs led to a reduction of DHE fluorescence in these cells and throughout the aged intestine (Figures 2D-E, S2A).

Loss of intestinal integrity can be assayed in living flies by monitoring the presence of non-absorbed dyes (e.g., FD&C blue No. 1) outside of the digestive tract after feeding [10, 11]. To determine whether *ndi1* can delay the onset of intestinal barrier dysfunction, we examined flies of different ages fed FD&C blue No. 1 for evidence of this dye outside of the digestive tract. The proportion of aged flies with dye outside of the intestine was significantly lower in flies with ISC/EB *ndi1* expression (Figure 2F). This was not a result of altered development, as adult onset induction of *ndi1* in ISCs/EBs, using the *5961GS* driver, was sufficient to decrease the proportion of flies with dye outside of the digestive tract with age (Figure 2G and S2B). Loss of intestinal integrity has been linked with a systemic increase in expression of immunity-related genes [10]. Hence, we assayed systemic expression levels of several anti-microbial peptides (AMPs) genes in *esgGAL4>ndi1* and control flies during aging. In line with decreased intestinal barrier dysfunction, flies that express *ndi1* in ISCs/EBs show significantly lower expression of multiple AMPs in whole bodies later in life (Figure 2H). Taken together, our findings show that ISC/EB-specific expression of *ndi1* leads to improved intestinal homeostasis during aging.

### *ndi1* expression in ISCs/EBs does not affect fertility or physical activity but changes sensitivity to some stresses

To gain further insight into intestinal *ndi1*-mediated longevity, we examined a number of physiological and behavioral parameters in long-lived *esgGAL4>ndi1* flies and controls. Neither male nor female flies that express *ndi1* in ISC/EBs showed consistent alterations to fertility (Figures S2C-G). Resistance to oxidative stress, assayed by survival under hyperoxia (80% O_2_), was similarly unaffected (Figure S2H), suggesting that ROS levels in the intestinal epithelium are not limiting for survival under severely hyperoxic conditions. Survival in elevated environmental temperatures (37°C) and water-only starvation showed considerable differences, with *ndi1* expressing flies showing significantly greater sensitivity to elevated temperatures (Figure S2I), and greater resistance to starvation (Figure S2J). These changes were not correlated with significant differences in either spontaneous locomotor activity per time of day (Figure S2K) or cumulative activity over 24-hour periods (Figure S2L). Together, these data indicate that intestinal *ndi1*-mediated longevity is not associated with a general increase in stress resistance or a decline in reproductive output.

### *ndi1* expression in ISCs/EBs stimulates feeding behavior

**Figure 3:**
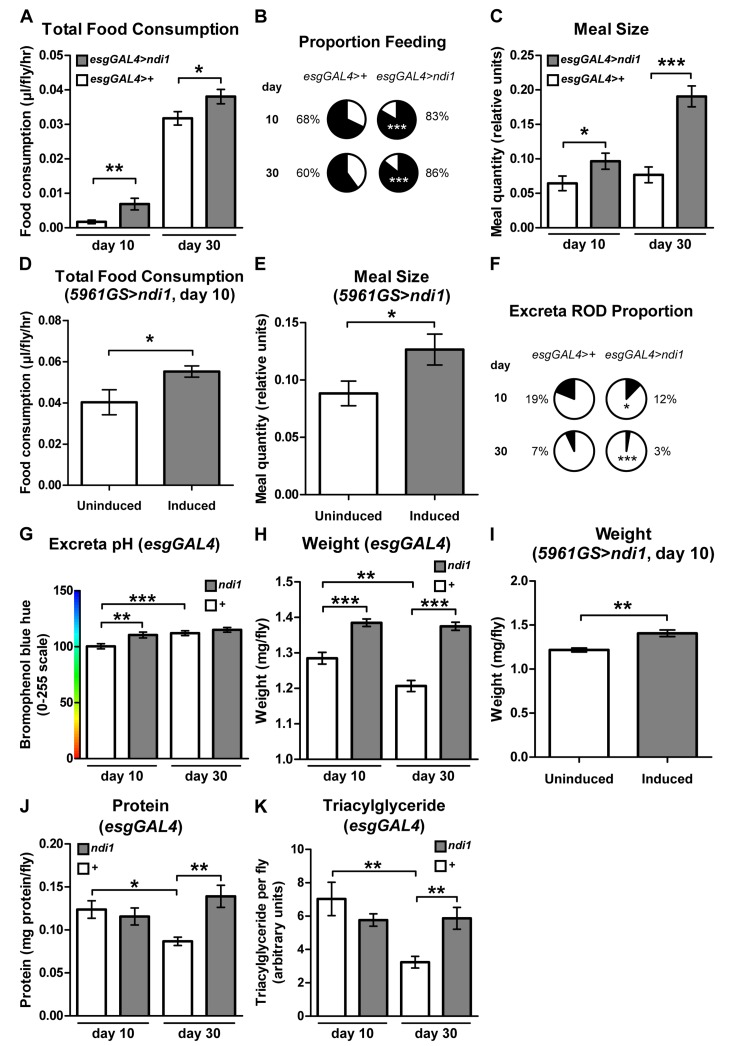
*ndi1* expression in the intestine stimulates feeding behavior (**A**) Analysis of total food consumption using a capillary feeding (CAFE) assay. Flies expressing *ndi1* in ISCs/EBs (*esgGAL4>ndi1*) consume significantly more food relative to controls (*esgGAL4>+*). (*p<0.05, **p<0.01, t test, 10 replicates per condition, 10 flies per replicate). (**B**) Analysis of feeding proportion using a colorimetric dye-tracking assay. Flies that express *ndi1* in ISCs/EBs (*esgGAL4>ndi1*) had a significantly greater proportion of flies that fed during the assay period relative to controls (*esgGAL4>+*). (***p<0.001, binomial test, approximately 90 flies per condition). (**C**) Analysis of meal size using a colorimetric dye-tracking assay (constitutive *ndi1* expression). Of those flies that ate during the assay period in (**B**), meal size was significantly greater in flies that express *ndi1* in ISCs/EBs (*esgGAL4>ndi1*) relative to controls (*esgGAL4>+*). (*p<0.05, ***p<0.001, t test, 50-95 flies that ate from B per condition). (**D**) Analysis of total food consumption using a CAFE assay in 5961GS>*ndi1* flies with or without RU486-mediated transgene induction. Ten days of induced *ndi1* expression in ISCs/EBs of adults by exposure to RU486 (0.5mg/l) increases total food consumption relative to uninduced controls. (*p<0.05, t test, 6 replicates per condition, 10 flies per replicate). (**E**) Analysis of meal size using a colorimetric dye-tracking assay in *5961GS>ndi1* flies with or without RU486-mediated transgene induction. Ten days of induced *ndi1* expression in ISCs/EBs of adults by exposure to RU486 (0.5mg/l) increases meal size relative to uninduced controls. (*p<0.05, t test, approximately 85 flies that ate during the assay period in Figure S3A). (**F**) Analysis of intestinal function by assaying excreta shape. The proportion of oblong deposits (RODs, Figure S3E, arrow) in excreta is significantly lower in deposits from flies expressing *ndi1* in ISCs/EBs (*esgGAL4>ndi1*) relative to controls (*esgGAL4>+*). (*p<0.05, ***p<0.001, binomial test, at least 180 deposits per condition). (**G**) Analysis of intestinal function by excreta pH. Flies expressing *ndi1* in ISCs/EBs (*“ndi1”, esgGAL4>ndi1*) had more alkaline excreta relative to controls (*“+”, esgGAL4>+*) at day 10 of adulthood in colorimetric analyses of excreta pH after feeding on BPB medium. (**p<0.01, ***p<0.001, t test, at least 180 deposits per condition). (**H**) Weights of flies with constitutive *ndi1* expression in ISCs/EBs as a function of age. Flies that express *ndi1* in ISCs/EBs (*“ndi1”, esgGAL4>ndi1*) are heavierthan isogenic controls (*“+”, esgGAL4>+*) and maintain their weight through day 30 of adulthood. (**p<0.01, **p<0.01, ***p<0.001, t test, 12 replicates per condition, 5 flies per replicate). (**I**) Weights of 5961GS>ndi1 flies with or without RU486-mediated transgene induction. Ten days of adulthood only induction of *ndi1* in ISCs/EBs by exposure to RU486 (0.5mg/l) is sufficient to significantly increase weight relative to uninduced controls. (**p<0.01, t test, 6 replicates per condition, 10 flies per replicate). (**J**) Protein content as a function of age. Protein content is significantly decreased in control flies (*“+”, esgGAL4>+*) at 30 days of adulthood, but is maintained in flies expressing *ndi1* in ISCs/EBs (*“ndi1”, esgGAL4>ndi1*). (*p<0.05, **p<0.01, t test, 4 replicates per condition, 5 flies per replicate). (**K**) Triacylglyceride content as a function of age. Thin-layer chromatography (Figure S3G) and densitometry for triacylglyceride content show a significant decrease in control flies (*“+”, esgGAL4>+*) at 30 days of age whereas flies that express *ndi1* in ISCs/EBs (*“ndi1”, esgGAL4>ndi1*) maintain triacylglyceride stores with age. (**p<0.01, t test, 5 replicates per condition, 5 flies per replicate).

A moderate reduction in food intake, dietary restriction (DR), can extend lifespan in diverse organisms, possibly by reducing the intake of specific nutrients [4]. To determine if a gross difference in food intake could play a role in *ndi1*-mediated longevity, we assayed feeding behavior in *esgGAL4>ndi1* flies and controls. Surprisingly, total food consumption, measured using a capillary feeding (CAFE) assay [27], revealed an overall increase in feeding in flies that express *ndi1* in ISCs/EBs at both young and aged time points (Figure 3A). An independent assay of feeding using a modified dye-tracking assay [28] was used to parse the feeding behavior into the proportion of flies that feed within the assay period and the meal size of flies that feed. Expression of *ndi1* in ISCs/EBs resulted in significant increases in both the proportion of flies that feed (Figure 3B) and their meal sizes (Figure 3C) in both young and aged flies. 24 hour activity profiles of *ndi1* expressing flies are similar to controls, suggesting that an altered activity at different times of day is not responsible for the increased feeding during the assay period (Figure S2K). Moreover, adult-onset expression of *ndi1* for 10 days in ISCs/EBs, using the *5961GS* driver, was sufficient to confer an increase in total food consumption (Figure 3D) and meal size (Figure 3E). The presence of the inducing drug itself had no significant effect on total feeding (Figure S3B) or meal size (Figure S3C).

To determine whether increased feeding was associated with alterations in defecation, we examined the material excreted by *esgGAL4>ndi1* flies and controls. Although *ndi1* expressing flies ate significantly more than controls, excreta number were not significantly different than controls, at both young and aged time points (Figure S3D). Recent work has shown that qualitative analysis of excreta can provide insight into intestinal transit and fluid homeostasis [29]. Specifically, flies that are starved for nutrients and fluids, as during times of high fecundity in females, were shown to have increased frequency of “reproductive oblong deposits” (RODs) and lower fecal pH. Closer examination of excreta shape of young and aged *esgGAL4>ndi1* flies showed a significant reduction in the frequency of RODs (Figures 3F and S3E) indicating improved fluid availability, less concentrated intestinal contents, and quicker intestinal transit [29]. Similarly, fecal pH analysis of flies maintained on bromophenol blue (BPB) containing diets showed less acidic fecal deposits in *ndi1* expressing flies at the young time point (Figure 3G), consistent with a quicker transit through the intestinal tract. Together, these findings indicate that expression of *ndi1* in ISCs/EBs in addition to improving tissue homeostasis, improves intestinal function.

Next, we set out to determine whether *esgGAL4>ndi1* flies show systemic physiological changes that are consistent with increased nutrient uptake. Whole body weight measurements indicated that *esgGAL4>ndi1* flies are heavier than controls, and maintain their weight during aging (Figure 3H). As with feeding behavior, adult-onset expression of *ndi1* for 10 days in ISCs/EBs, using the *5961GS* driver was sufficient to confer an increase in body weight (Figure 3I), and this was not a result of the inducing drug itself (Figure S3F). Moreover, aged *esgGAL4>ndi1* flies display increased protein levels and triglyceride stores relative to controls at aged timepoints (Figures 3J-K, S3G). Unlike triglycerides, levels of glycogen declined similarly in both *ndi1* expressing flies and controls with age (Figure S3H).

### *ndi1* expression in ISCs/EBs alters systemic metabolic signaling pathways

**Figure 4:**
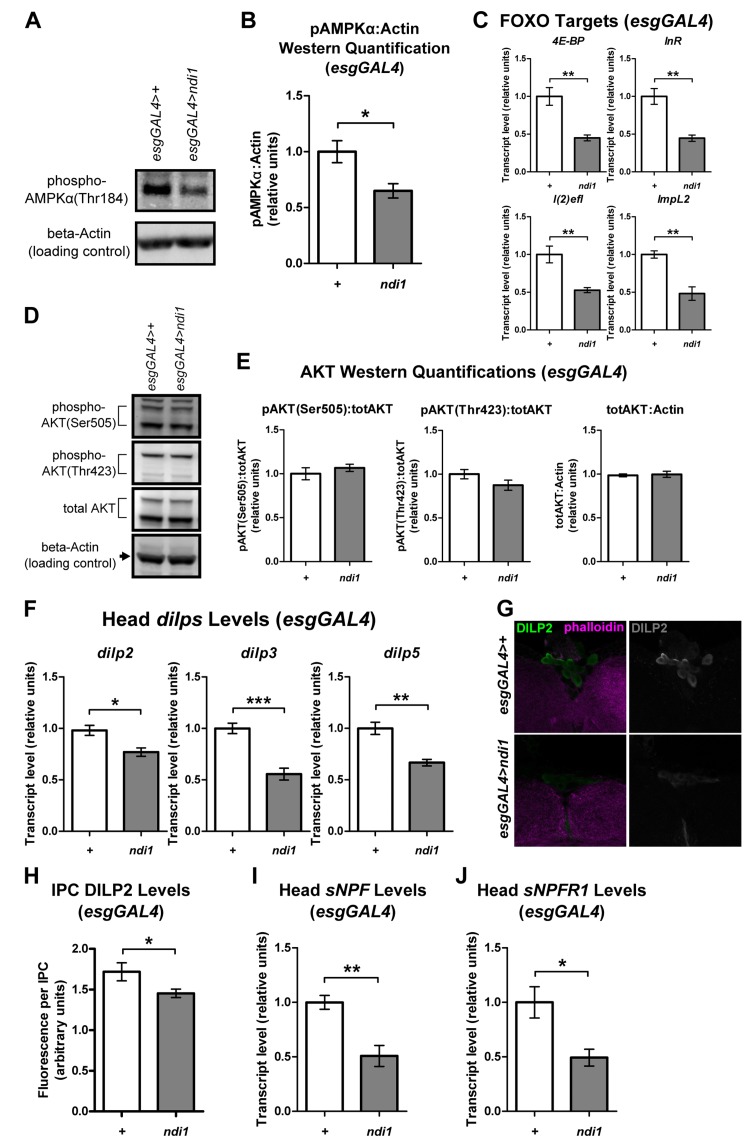
*ndi1* expression in the intestine produces alterations in systemic metabolic signaling pathways (**A-B**) Western blot (A, Figure S4A) and densitometric analysis (**B**) of AMPKα phosphorylation at Thr184. AMPKα phosphorylation (normalized to a loading control, beta-Actin)is significantly decreased in flies that express *ndi1* in ISCs/EBs (“*ndi1*”, *esgGAL4>ndi1*), relative to isogenic controls (“*+*”, *esgAL4>+*) at day 10 of adulthood. (*p<0.05, t test, 5 replicates per condition, 15 flies per replicate). (**C**) Transcript levels of downstream targets of dFOXO. *4E-BP, InR, l(2)efl*, and *ImpL2* transcript levels are significantly lower in flies that express *ndi1* in ISCs/EBs (“*ndi1*”, *esgGAL4>ndi1*) relative to isogenic controls (“*+*”, *esgAL4>+*) at day 10 of adulthood. (**p<0.01, t test, 5 replicates per condition, 5 flies per replicate). (**D-E**) Western blot (D, Figure S4B) and densitometric analysis (**E**) of AKT phosphorylation at Ser505 or Thr423, and total AKT levels. AKT phosphorylation (normalized to total AKT) is not altered in flies that express *ndi1* in ISCs/EBs (“*ndi1*”, *esgGAL4>ndi1*) relative to isogenic controls (“*+*”, *esgAL4>+*) at day 10 of adulthood. Total AKT levels (normalized to beta-Actin) are also unchanged. (n.s., t test, 5 replicates per condition, 5 flies per replicate). (**F**) Transcript levels of head *dilp* genes. *dilp2, dilp3, and dilp5* transcript levels from heads of flies that express *ndi1* in ISCs/EBs (“*ndi1*”, *esgGAL4>ndi1*) are significantly lower than those of controls (“*+*”, *esgAL4>+*) at day 10 of adulthood. (*p<0.05, **p<0.01, ***p<0.001, t test, 5 replicates per condition, 30 heads per replicate). (**G-H**) Immunofluorescence staining (**G**) and quantification (**H**) of DILP2 levels in insulin producing cells (IPCs). DILP2 fluorescence in IPCs of flies that express *ndi1* in ISCs/EBs (“*ndi1*”, *esgGAL4>ndi1*) are significantly lower compared to that of controls (“*+*”, *esgAL4>+*) at day 10 of adulthood. (*p<0.05, t test, at least 170 IPCs from 12 different brains). (**I-J**) Transcript levels of *sNPF* (**I**) and its cognate receptor, sNPFR1 (**J**) in heads. Flies that express *ndi1* in ISCs/EBs (“*ndi1*”, *esgGAL4>ndi1*) have significantly lower sNPF and sNPFR1 transcript levels in heads than controls (“*+*”, *esgAL4>+*) at day 10 of adulthood. (*p<0.05, **p<0.01, t test, 4-5 replicates per condition, 30 heads per replicate).

We set out to further characterize the physiology of long-lived *ndi1* flies by examining steady state effects of *ndi1* expression on systemic nutrient sensitive pathways. AMP-activated protein kinase (AMPK) is a crucial metabolic gauge that is activated by low cellular energy status [16]. Since expression of *ndi1* in ISCs/EBs stimulates feeding, we reasoned that these flies may show reduced systemic AMPK activity. Indeed, Western blots specific for phosphorylated AMPKrevealed significantly decreased phosphorylation at Thr184 in whole bodies of *esgGAL4>ndi1* flies relative to controls (Figures 4A-B and S4A). AMPK activation has been shown to stimulate sirtuin1 (SIRT1) activity, which deacetylates FOXO and increases its transcriptional activity [30]. Consistent with this, systemic dFOXO transcriptional activity, assayed by measuring transcript levels of multiple direct downstream targets of dFOXO in whole bodies, was significantly decreased in *esgGAL4>ndi1* flies (Figure 4C).

FOXO activity is independently regulated by a number of different signaling pathways, including the insulin/insulin-like growth factor signaling (IIS) pathway [31]. To determine whether the observed decrease in dFOXO activity in *esgGAL4>ndi1* flies was also associated with an increase in systemic IIS, we assayed the activation state of the direct mediator of FOXO activity in the IIS pathway, phosphoinositide-3-OH-kinase-dependent serine/threonine protein kinase (AKT). In whole bodies of *esgGAL4>ndi1* flies the phosphorylation of AKT was not significantly affected at either the IIS phosphorylation site (Thr423) or the TORC2 phosphorylation site (Ser505), nor was there a difference in total AKT protein levels (Figures 4D-E and S4B). Similarly, Western blot analysis of S6K, a component of the TOR pathway revealed no significant changes in phosphorylation at the TORC1 target residue (Thr398) or in total S6K levels in whole bodies of *ndi1* expressing flies (Figures S4C-D). Thus, the observed decrease in FOXO transcriptional activity in *esgGAL4>ndi1* flies is not associated with alterations in systemic IIS/TOR pathway activities.

*ndi1* expression affects both feeding behavior and longevity, both of which have been shown to be regulatable by *Drosophila* insulin-like peptides (*dilp*s) signaling [32-34]. Therefore, we checked the expression levels of several *dilp*s to determine if *dilp* levels in heads of *esgGAL4>ndi1* flies were altered. Transcript levels in heads of *dilp2*,*dilp3* and *dilp5*, that are expressed in the insulin producing cells (IPCs) upon feeding, were significantly decreased in *esgGAL4>ndi1* flies (Figure 4F) while the transcript level of *dilp1*, which is not expressed in adult heads [32], was not altered significantly (Figure S4E). Direct quantification of DILP2 protein levels in IPCs by immunofluorescence showed a similar decrease in DILP2 fluorescence in IPCs of *esgGAL4>ndi1* flies (Figure 4G and 4H). The *Drosophila* ortholog of mammalian neuropeptide Y, short neuropeptide F (sNPF), is expressed in the nervous system and regulates food intake [35] and along with its cognate receptor, *sNPFR1*, regulates the expression of *dilps* in the fly brain [36]. To determine if decreased *dilp* transcription in heads of *esgGAL4>ndi1* flies is linked to altered sNPF/sNPFR1 expression, we measured transcript levels of *sNPF* and *sNPFR1* in heads. In line with *dilp* transcript levels in heads, both *sNPF* and *sNPFR1* transcript levels were decreased in heads of *esgGAL4>ndi1* flies relative to controls (Figure 4I and 4J).

## DISCUSSION

A decline in mitochondrial activity has been implicated in multiple degenerative diseases of aging [2]. These findings raise the intriguing possibility that strategies to stimulate mitochondrial activity during aging may delay the onset of pathology and extend healthspan. In support of this idea, we recently reported that overexpression of the fly PGC-1 homolog, *dPGC-1*, in ISC lineages is sufficient to preserve intestinal homeostasis during aging and extend fly lifespan [11]. However, due to the extensive interactions that PGC-1 has with multiple aspects of metabolism [12], the possibility persists that endogenous *dPGC-1* interactions, other than its role as a regulator of mitochondrial activity, play a role in the cellular and/or organismal phenotypes that we observed. Unlike *dPGC-1, ndi1* is exogenous, from a different kingdom, with no known homologs in animals, so any changes that result from *ndi1* expression can reasonably be expected to be from the function of *ndi1* as an NADH dehydrogenase. A previous study reported that ubiquitous expression of *ndi1* using a constitutive driver line can increase fly lifespan [13]. However, studies of the genetics of aging and lifespan determination are prone to confounding effects due to uncontrolled differences in genetic background between test and control lines [37]. Using an inducible gene expresion system, which eliminates this issue, we failed to observe lifespan extension upon ubiquitous expression, but instead observed that neuron-specific expression of *ndi1* can extend lifespan [15]. In the present study, we have extended this approach and show that expression of *ndi1* in adult intestinal stem and progenitor cells can reduce whole tissue ROS levels, improve tissue homeostasis, delay the onset of intestinal barrier dysfunction, and extend the lifespan of flies. Therefore, a major conclusion of this study is that an increase in mitochondial NADH dehydrogenase activity alone in ISCs/EBs can delay both tissue and organismal aging, possibly by limiting pro-proliferative ROS levels in the intestinal epithelium.

Long-lived flies expressing *ndi1* in ISCs/EBs have behavioral, physiological, and biochemical correlates of increased nutrition, showing increased feeding, weight, metabolic stores, and decreased systemic activation of AMPK. Importantly, *ndi1*-mediated weight gain can be observed upon adult-onset expression in ISCs/EBs. Moreover, both increased sensitivity to elevated temperatures, and resistance to starvation of the long-lived flies are wholly consistent with larger flies (with lower surface-to-mass ratios) and improved nutrient absorption and storage. Further studies using radioactive tracers of specific nutrients may provide clues as to whether increased total caloric uptake or differential absorption of specific nutrients play a role in the increased longevity of *ndi1* expressing flies. Regardless of whether total caloric intake or absorbed nutrient composition plays a bigger role, one indication that improved nutrition plays a role in increasing lifespan is the ability of flies expressing *ndi1* in ISCs/EBs to retain body weight and metabolic stores with age.

Forkhead Box-O (FOXO) transcription factors, inhibited by IIS, have been implicated in metabolic homeostasis and lifespan determination [38]. Indeed, adult-onset and tissue-restricted overexpression of the single *Drosophila* FOXO ortholog (*dFOXO*) can increase longevity [39, 40]. Yet, the relationships between IIS, FOXO activity and organismal health are not straightforward. Reduced IIS in mammals results in diabetes, whose associated pathologies shorten lifespan, and aged flies display markers of impaired IIS, including dFOXO activation, which are tightly linked to impending death [10, 41]. In the current study, we show that long-lived flies, expressing *ndi1* in ISCs/EBs, show reduced expression of multiple dFOXO target genes in whole bodies. However, reduced dFOXO activity was not associated with alterations in AKT activation indicating that systemic IIS activity is not altered. Examination of *dilp* levels in *ndi1* expressing flies revealed low transcript levels of head *dilps*. Therefore, *ndi1* expression in ISCs/EBs may result in uncoupling of DILP signaling from nutritional status. Although the spatially and temporally dynamic nature of feeding and nutrition signaling make definitive interpretations difficult, one possibility that is consistent with our findings is that feeding suppresses AMPK activity, leading to decreased FOXO activity and *sNPF*/*sNPFR1* transcript levels. Without a corresponding increase in DILP levels to inhibit feeding, however, the flies remain in a permissive state for feeding, and even with reduced sNPF/sNPFR1 signaling, eat more.

How do we reconcile our findings with previous work reporting that reduced IIS and/or FOXO activation prolongs lifespan in *Drosophila* [5]? Our observation that long-lived flies expressing *ndi1* in ISCs/EBs show reduced expression of *dilps* in heads and DILP2 levels in IPCs may provide some insight. Reduced expression of *dilp2* has been consistently associated with increased lifespan in multiple gentotypes in studies from different laboratories [32, 33, 36, 39, 42, 43]. Moreover, deletion of the neurosecretory cells that produce *dilp2, 3*, and *5* produces phenotypes that overlap with ISC/EB expression of *ndi1*, including resistance to starvation stress, sensitivity to heat stress, and increased lifespan [32]. Uncovering the mechanism by which *ndi1* expression in ISCs/EBs results in altered expression of *dilps* could provide important insights into the role of somatic stem cells in the regulation organismal lifespan, metabolism and behavior. Regardless of the underlying mechanisms, our findings demonstrate that providing exogenous NADH dehydrogenase activity in ISCs/EBs is an attractive strategy to delay markers of intestinal aging and prolong healthy lifespan in fruit flies. Given that *ndi1* can be functionally expressed in mammalian cells [44-46], and does not cause an immune response [47], expression of *ndi1* in mammalian stem cells may provide a strategy to similarly improve tissue homeostasis and delay the onset of aging.

## EXPERIMENTAL PROCEDURES

Unless otherwise specified, mated female flies were used for experimental analyses. For full descriptions of methods used in this study, please see Supporting Information Materials and Methods.

### Fly lines, culture, and genotypes

*UAS-ndi1* lines [15] were backcrossed 10 times into a *w^1118^* background. *TIGS-2* line was provided by L. Seroude, *5961GS* was provided by H. Jasper, and esgGAL4 was provided by A. Christiansen. Culturing of flies and measurements of lifespan were performed as previously described [11]. See Supporting Information Materials and Methods for details.

Genotypes:*TIGS>+*:*+;+;TIGS-2/+,TIGS>ndi1*:*+;UAS-ndi1/+;TIGS-2/+,esgGAL4>+*:*+;esgGAL4,UAS-gfp/+;+,esgGAL4>ndi1*:*+;esgGAL4,UAS-gfp/UAS-ndi1;+,5961GS>+*:*+;5961GS,UAS-gfp/+;+,5961GS>ndi1*:*+;5961GS,UAS-gfp/UAS-ndi1;+,5966GS>+*:*+;5966GS/+;+,5966GS>ndi1*:*+;5966GS/UAS-ndi1;+*

### Feeding Assays

Capillary feeding (CAFE) assays and dye-tracking assays were adapted from previously described protocols with slight modifications [27, 28]. See Supporting Information Materials and Methods for details.

### Molecular Biology and Physiology

Protocols and reagents used for complex I and NDI1 activity assays, immunofluorescence staining, ROS staining, quantitative real-time polymerase chain reaction (qRT-PCR), intestinal barrier and transit assays, and measurements of fertility, stress resistance, spontaneous activity, weights, and metabolites are provided in Supporting Information Materials and Methods.

### Statistical Analyses

Unless otherwise indicated, significance was determined using a two-tailed, unpaired *t* test from at least three independent experiments and expressed as p values. All error bars reflect standard error of the mean.

## SUPPLEMENTAL DATA





## References

[R1] Jones DL, Rando TA (2011). Emerging models and paradigms for stem cell ageing. Nature cell biology.

[R2] Green DR, Galluzzi L, Kroemer G (2011). Mitochondria and the autophagy-inflammation-cell death axis in organismal aging. Science.

[R3] Jasper H, Jones DL (2010). Metabolic regulation of stem cell behavior and implications for aging. Cell metabolism.

[R4] Piper MD, Bartke A (2008). Diet and aging. Cell metabolism.

[R5] Alic N, Partridge L (2011). Death and dessert: nutrient signalling pathways and ageing. Current opinion in cell biology.

[R6] Micchelli CA, Perrimon N (2006). Evidence that stem cells reside in the adult Drosophila midgut epithelium. Nature.

[R7] Ohlstein B, Spradling A (2006). The adult Drosophila posterior midgut is maintained by pluripotent stem cells. Nature.

[R8] Choi NH, Kim JG, Yang DJ, Kim YS, Yoo MA (2008). Age-related changes in Drosophila midgut are associated with PVF2, a PDGF/VEGF-like growth factor. Aging cell.

[R9] Biteau B, Hochmuth CE, Jasper H (2008). JNK activity in somatic stem cells causes loss of tissue homeostasis in the aging Drosophila gut. Cell stem cell.

[R10] Rera M, Clark RI, Walker DW (2012). Intestinal barrier dysfunction links metabolic and inflammatory markers of aging to death in Drosophila. Proceedings of the National Academy of Sciences of the United States of America.

[R11] Rera M, Bahadorani S, Cho J, Koehler CL, Ulgherait M, Hur JH, Ansari WS, Lo T, Jones DL, Walker DW (2011). Modulation of longevity and tissue homeostasis by the Drosophila PGC-1 homolog. Cell metabolism.

[R12] Lin J, Handschin C, Spiegelman BM (2005). Metabolic control through the PGC-1 family of transcription coactivators. Cell Metab.

[R13] Sanz A, Soikkeli M, Portero-Otin M, Wilson A, Kemppainen E, McIlroy G, Ellila S, Kemppainen KK, Tuomela T, Lakanmaa M, Kiviranta E, Stefanatos R, Dufour E (2010). Expression of the yeast NADH dehydrogenase Ndi1 in Drosophila confers increased lifespan independently of dietary restriction. Proceedings of the National Academy of Sciences of the United States of America.

[R14] Cho J, Hur JH, Graniel J, Benzer S, Walker DW (2012). Expression of yeast NDI1 rescues a Drosophila complex I assembly defect. PloS one.

[R15] Bahadorani S, Cho J, Lo T, Contreras H, Lawal HO, Krantz DE, Bradley TJ, Walker DW (2010). Neuronal expression of a single-subunit yeast NADH-ubiquinone oxidoreductase (Ndi1) extends Drosophila lifespan. Aging cell.

[R16] Hardie DG, Ross FA, Hawley SA (2012). AMPK: a nutrient and energy sensor that maintains energy homeostasis. Nature reviews Molecular cell biology.

[R17] Rera M, Azizi MJ, Walker DW (2013). Organ-specific mediation of lifespan extension: More than a gut feeling?. Ageing research reviews.

[R18] Poirier L, Shane A, Zheng J, Seroude L (2008). Characterization of the Drosophila gene-switch system in aging studies: a cautionary tale. Aging cell.

[R19] de Vries S, Grivell LA (1988). Purification and characterization of a rotenone-insensitive NADH:Q6 oxidoreductase from mitochondria of Saccharomyces cerevisiae. European journal of biochemistry / FEBS.

[R20] Osterwalder T, Yoon KS, White BH, Keshishian H (2001). A conditional tissue-specific transgene expression system using inducible GAL4. Proceedings of the National Academy of Sciences of the United States of America.

[R21] Roman G, Endo K, Zong L, Davis RL (2001). P[Switch], a system for spatial and temporal control of gene expression in Drosophila melanogaster. Proc Natl Acad Sci U S A.

[R22] Biteau B, Karpac J, Supoyo S, Degennaro M, Lehmann R, Jasper H (2010). Lifespan extension by preserving proliferative homeostasis in Drosophila. PLoS genetics.

[R23] Mathur D, Bost A, Driver I, Ohlstein B (2010). A transient niche regulates the specification of Drosophila intestinal stem cells. Science.

[R24] Buchon N, Broderick NA, Chakrabarti S, Lemaitre B (2009). Invasive and indigenous microbiota impact intestinal stem cell activity through multiple pathways in Drosophila. Genes & development.

[R25] Hochmuth CE, Biteau B, Bohmann D, Jasper H (2011). Redox regulation by Keap1 and Nrf2 controls intestinal stem cell proliferation in Drosophila. Cell stem cell.

[R26] Owusu-Ansah E, Banerjee U (2009). Reactive oxygen species prime Drosophila haematopoietic progenitors for differentiation. Nature.

[R27] Ja WW, Carvalho GB, Mak EM, de la Rosa NN, Fang AY, Liong JC, Brummel T, Benzer S (2007). Prandiology of Drosophila and the CAFE assay. Proceedings of the National Academy of Sciences of the United States of America.

[R28] Wong R, Piper MD, Wertheim B, Partridge L (2009). Quantification of food intake in Drosophila. PloS one.

[R29] Cognigni P, Bailey AP, Miguel-Aliaga I (2011). Enteric neurons and systemic signals couple nutritional and reproductive status with intestinal homeostasis. Cell metabolism.

[R30] Canto C, Gerhart-Hines Z, Feige JN, Lagouge M, Noriega L, Milne JC, Elliott PJ, Puigserver P, Auwerx J (2009). AMPK regulates energy expenditure by modulating NAD+ metabolism and SIRT1 activity. Nature.

[R31] Samuel VT, Shulman GI (2012). Mechanisms for insulin resistance: common threads and missing links. Cell.

[R32] Broughton SJ, Piper MD, Ikeya T, Bass TM, Jacobson J, Driege Y, Martinez P, Hafen E, Withers DJ, Leevers SJ, Partridge L (2005). Longer lifespan, altered metabolism, and stress resistance in Drosophila from ablation of cells making insulin-like ligands. Proceedings of the National Academy of Sciences of the United States of America.

[R33] Bai H, Kang P, Tatar M (2012). Drosophila insulin-like peptide-6 (dilp6) expression from fat body extends lifespan and represses secretion of Drosophila insulin-like peptide-2 from the brain. Aging cell.

[R34] Geminard C, Rulifson EJ, Leopold P (2009). Remote control of insulin secretion by fat cells in Drosophila. Cell metabolism.

[R35] Lee KS, You KH, Choo JK, Han YM, Yu K (2004). Drosophila short neuropeptide F regulates food intake and body size. The Journal of biological chemistry.

[R36] Lee KS, Kwon OY, Lee JH, Kwon K, Min KJ, Jung SA, Kim AK, You KH, Tatar M, Yu K (2008). Drosophila short neuropeptide F signalling regulates growth by ERK-mediated insulin signalling. Nature cell biology.

[R37] Partridge L, Gems D (2007). Benchmarks for ageing studies. Nature.

[R38] Piper MD, Selman C, McElwee JJ, Partridge L (2008). Separating cause from effect: how does insulin/IGF signalling control lifespan in worms, flies and mice?. Journal of internal medicine.

[R39] Hwangbo DS, Gershman B, Tu MP, Palmer M, Tatar M (2004). Drosophila dFOXO controls lifespan and regulates insulin signalling in brain and fat body. Nature.

[R40] Giannakou ME, Goss M, Junger MA, Hafen E, Leevers SJ, Partridge L (2004). Long-lived Drosophila with overexpressed dFOXO in adult fat body. Science.

[R41] Morris SN, Coogan C, Chamseddin K, Fernandez-Kim SO, Kolli S, Keller JN, Bauer JH (2012). Development of diet-induced insulin resistance in adult Drosophila melanogaster. Biochimica et biophysica acta.

[R42] Wang MC, Bohmann D, Jasper H (2005). JNK extends life span and limits growth by antagonizing cellular and organism-wide responses to insulin signaling. Cell.

[R43] Gronke S, Clarke DF, Broughton S, Andrews TD, Partridge L (2010). Molecular evolution and functional characterization of Drosophila insulin-like peptides. PLoS genetics.

[R44] Seo BB, Wang J, Flotte TR, Yagi T, Matsuno-Yagi A (2000). Use of the NADH-quinone oxidoreductase (NDI1) gene of Saccharomyces cerevisiae as a possible cure for complex I defects in human cells. The Journal of biological chemistry.

[R45] Seo BB, Kitajima-Ihara T, Chan EK, Scheffler IE, Matsuno-Yagi A, Yagi T (1998). Molecular remedy of complex I defects: rotenone-insensitive internal NADH-quinone oxidoreductase of Saccharomyces cerevisiae mitochondria restores the NADH oxidase activity of complex I-deficient mammalian cells. Proceedings of the National Academy of Sciences of the United States of America.

[R46] Santidrian AF, Matsuno-Yagi A, Ritland M, Seo BB, LeBoeuf SE, Gay LJ, Yagi T, Felding-Habermann B (2013). Mitochondrial complex I activity and NAD+/NADH balance regulate breast cancer progression. The Journal of clinical investigation.

[R47] Marella M, Seo BB, Flotte TR, Matsuno-Yagi A, Yagi T (2011). No immune responses by the expression of the yeast Ndi1 protein in rats. PloS one.

