# Supplementary material for: Increased longevity mediated by yeast NDI1 expression in Drosophila intestinal stem and progenitor cells
Source: Aging (Albany NY). 2013 Sep 6;5(9):662–81. doi: 10.18632/aging.100595 (PMC3808699; doi:10.18632/aging.100595)
Supplement: Supplementary file 1 [file aging-05-662-s001.doc]

### SI Materials and Methods

**Genotypes**

Genotypes: *TIGS>+*: *+;+;TIGS-2/+, TIGS>ndi1*: *+;UAS-ndi1/+;TIGS-2/+, esgGAL4>+*: *+;esgGAL4,UAS-gfp/+;+, esgGAL4>ndi1*: *+;esgGAL4,UAS-gfp/UAS-ndi1;+, 5961GS>+*: *+;5961GS,UAS-gfp/+;+, 5961GS>ndi1*: *+;5961GS,UAS-gfp/UAS-ndi1;+, 5966GS>+*: *+;5966GS/+;+, 5966GS>ndi1*: *+;5966GS/UAS-ndi1;+*

### Fly Media

Unless otherwise noted solid food media provided to flies contained 1% agar, 3% yeast, 1.9% sucrose, 3.8% dextrose, 9.1% cornmeal, 1.5% methylparaben, and 1% acid mix wt/vol. For RU486 and control media, a stock solution of RU486 (Cayman Chemicals, Ann Arbor, MI, USA) or ethanol was mixed into the media to specified concentrations after cooling to approximately 65ºC.

### Lifespan Analysis

Flies that eclosed over a 24-36 hour period were collected and allowed to mate for approximately 60 hours. Female or male flies were collected under light nitrogen-induced anesthesia and maintained at a density of 30 flies per vial in a humidified, temperature-controlled (25ºC) incubator with a 12 hour light-dark cycle. Flies were transferred to new vials every 2-3 days and scored for death.

**Mitochondrial Isolation**

Fly guts were dissected on ice on ice-cold 1XPBS, and mitochondria were purified by differential centrifugation. Dissected guts were homogenized in chilled mitochondrial isolation medium (MIM, 250 mM sucrose, 10 mM Tris (pH 7.4), 0.15 mM MgCl_2_) and debris was pelleted by centrifugation (500×g, 5 min at 4°C). Mitochondria were pelleted by centrifugation (5,000×g, 5 min at 4°C) and stored at −80°C.

**NADH:Ubiquinone Oxidoreductase Activity Assay**

Prepared mitochondria were resuspended in MIM and added to 150 µl of a colorimetric complex I activity assay buffer (1× PBS, 3.5 g/l BSA, 0.2 mM NADH, 0.24 mM KCN, 60 µM DCIP, 70 µM decylubiquinone, 25 mM antimycin A). NADH:ubiquinone oxidoreductase activity was monitored as a drop in DCIP absorbance at 600 nm using an Epoch microplate spectrophotometer (BioTek, Winooski, VT, USA). Flavone or rotenone insensitive activity was measured as the difference in DCIP reduction in the presence of flavone (0.4 mM) or rotenone (2 µM) in the assay buffer, and baseline activity in an assay buffer that contained both inhibitors. All reported activities are normalized to citrate synthase activity.

### Citrate Synthase Activity Assay

Prepared mitochondria were diluted into MIM and added to 150 µl of a colorimetric citrate synthase activity assay buffer (50 mM Tris (pH 8.0), 0.1 mM 5,5′-dithiobis-(2-nitrobenzoic acid) (DTNB), 0.3 mM acetyl-CoA, 1 mM oxaloacetic acid). Citrate synthase activity was measured as an increase in DTNB absorbance at 412 nm using an Epoch microplate spectrophotometer (BioTek).

### Feeding Assays

Capillary feeding (CAFE) assays were performed as previously described with modifications. Briefly, 10 flies were placed in vials with wet tissue paper as a water source and a capillary food source (5% sucrose, 5% yeast extract, 2.5% FD&C Blue No. 1 (SPS Alfachem, Lexington, MA, USA)). Feeding was monitored from approximately 3 hours after lights on until lights off, with capillaries being replaced and feeding amounts recorded every 2-3 hours.

Dye tracking assays were performed as described previously with slight modifications. Approximately 30 flies were placed in vials with solid media supplemented with 2.5% dye (FD&C Blue No. 1) from 3 hours after lights on until 5.5 hours after lights on at 25°C. Possibly due to the low yeast concentration in our standard medium (0.3% yeast), flies required approximately 2.5 hours to show appreciable feeding. Even after 2.5 hours, significant proportions of flies still had not eaten (determined as described below), suggesting that our assay conditions were not saturating. Flies were frozen, decapitated, and homogenized separately in 200µl H2O. Cell debris was pelleted via centrifugation, and absorbance (629nm) of a 1:2.5 dilution of the supernatant was used to determine whether the fly ate (flies were categorized as having eaten if they had A629nm greater than 110% of the absorbance outside of the dye absorption range, at 800nm), and if so, the relative meal size (A629-A800).

**qRT-PCR**

RNA isolation, cDNA synthesis, and quantitative PCR were performed on whole flies or dissected body parts as previously described . Briefly, total RNA was extracted using TRIzol reagent (Life Technologies, Grand Island, NY, USA) following manufacturer protocols. Samples were treated with DNase I and used for cDNA synthesis using a Fermentas RevertAid First Strand cDNA Synthesis Kit (Thermo Fisher Scientific, Waltham, MA, USA). Quantitative RT-PCR was performed using a Power SYBR Green PCR Master Mix (Life Technologies) in a 45 cycle reaction in a Applied Biosystems 7300 Thermal Cycler (Life Technologies). Amplicons of actin5C were used as a reference for normalization. Primer sequences: l(2)efl: CAGACGCGTTTATCCAAGTG, ATCCCACCAGTCACGGAAC, dilp1: GCTTTAATACGCTGCCAAGG, CGGATCCGTACAGATTGGTT, dilp2: ATCCCGTGATTCCACACAAG, GCGGTTCCGATATCGAGTTA, dilp3: AGAGAACTTTGGACCCCGTGA, TGAACCGAACTATCACTCAAC, dilp5: GCCTTGATGGACATGCTGA, TCATAATCGAATAGGGCCCAAG, sNPF: CCCGAAAACTTTTAGACTCA, TTTTCAAACATTTCCATCGT, sNPFR1: CTGGCCATATCGGACCTACT, GGCCAGTACTTGGACAGGAT, actin5c, drosomycin, diptericin, drosocin, InR, 4E-BP, ImpL2 primers were previously described .

**Immunofluorescence and Cell Quantification**

Fixation and staining of Drosophila midguts were carried out as previously described . Midguts were co-labeled with chicken anti-GFP (1:5000, #GFP-1010, Aves Labs, Tigard, Oregon, USA) and rabbit anti-phospho-histone H3 (1:200, #06-570, EMD Millipore, Darmstadt, Germany) before mounting in Vectashield mounting medium (Vector Laboratories, Burlingame, CA, USA) containing 4’,6-diamidino-2-phenylindole (DAPI). Images of the posterior midgut (approximately 250 μm anterior to the pyloric ring) were acquired with a 20x objective on a Zeiss LSM 780 confocal microscope. Cells that stained for either GFP, phospho-histone H3 (pHH3^+^), or DAPI were counted manually from at least 22 samples per treatment group. For GFP^+^ counts, One-way ANOVA was used to determine statistical significance; means of each treatment were compared using Tukey’s post hoc test. To determine significant differences in the median number of pHH3+ cells per posterior midgut, a Kruskall-Wallis test was used followed by a Dunn’s post hoc test.

### Dihydroethidium (DHE) Staining

ROS levels were detected in live tissues as previously described . Briefly, guts were dissected in ice-cold Schneider’s medium (Caisson Labs, North Logan, UT, USA) and briefly fixed in 4% formaldehyde in Schneider’s medium for 3 minutes at room temperature. Guts were washed twice in Schneider’s medium for 30 seconds and stained in 50μM DHE (Life Technologies) in Schneider’s medium for 7 minutes at room temperature in a light protected chamber. Samples were washed three times for 5 minutes in Schneider’s medium at room temperature, mounted in Prolong Gold with DAPI (Life Technologies), and imaged immediately at 100X magnification, 200–500 µm anterior to the pylorus.

**Intestinal Barrier Dysfunction**

Flies were tested in groups of approximately 30 per vial, as previously described . Briefly, flies were transferred to solid media supplemented with 2.5% dye (FD&C Blue No. 1) for 9 hours and then checked visually under a dissecting microscope for evidence of dye outside of the gut.

### Weight

Flies were weighed in groups of 5 or 10 in pre-weighed microcentrifuge tubes, using an analytical scale (Torbal, Clifton, NJ, USA).

### Triacylglyceride

Lipids were extracted from whole flies in a chloroform:methanol:water solution (2:5:1 by volumes) and nonpolar lipids were separated on thin layer chromatography plates (Analtech, Newark, DE, USA) with a n-hexane:diethylether:glacial acetic acid solution (70:30:1 by volumes). Plates were air-dried, stained (0.2% Amido Black 10B in 1M NaCl), and lipid bands were quantified by photo densitometry using ImageJ as described .

### Glycogen and Protein

Decapitated flies were homogenized in ice-cold water, cleared and used for glycogen quantification using a Glycogen Colorimetric/Fluorometric Assay Kit (BioVision, Milpitas, CA, USA) following manufacturer protocols. Protein content was quantified using a µBCA kit (Thermo Fisher Scientific) following manufacturer protocols using the same clarified homogenates.

**Spontaneous Activity**

Spontaneous activity was measured as previously described . Briefly, flies were placed in a Drosophila activity meter (TriKinetics, Waltham, MA, USA) in vials at a density of 30 flies per vial. Movements were recorded continuously under normal culturing conditions for at least 24 hours on a 12 hour light-dark cycle.

**Fertility**

Virgin female flies were collected over a 24-hour period and matured for an additional 60 hours before mating to *w^1118^* flies, either for a single day or continuously. Flies were transferred to new vials every 1-2 days and fertility was measured over the entire lifespan by counting total adult progeny that had eclosed from the old vials after 2 weeks at 25ºC. Continuously mated flies were maintained at a density of 10 females and 10 males per vial and single day mated flies were maintained at a density of 10 females per vial.

### Stress Resistance

All stress assays were performed with mated female flies 10 days post eclosion at a density of 25-30 flies per vial. For hyperoxia resistance, flies were maintained in a humidified chamber maintained at 85% O_2_ and survival was assayed at least once per day. For heat stress resistance, flies were maintained in a humidified 37°C chamber and survival was scored every 2 hours. For wet starvation, flies were maintained on water only medium (1% agar in ddH_2_O wt/wt) and maintained in a 25°C incubator with 12 hour light-dark cycles. Survival was scored multiple times per day.

### Fecal Deposit Number, Shape, and pH

Excreta assays were performed as previously described with modifications. Flies were maintained on solid media supplemented with 0.5% bromophenol blue, sodium salt (BPB, Sigma-Aldrich, St. Louis, MO, USA) for 24 hours before the assay. Groups of 10 flies were maintained in 60X15mm petri dishes with 2ml BPB medium for 72 hours, and the center of the lid was photographed and analyzed using ImageJ for fecal number, shape, and hue.

### Western Blot

Western blot analyses were performed using standard procedures for Polyvinylidene difluoride (PVDF) membranes using the following antibodies and dilutions: phospho-AMPKα (Thr184) (1:1000, #2535, Cell Signaling Technology, Danvers, MA, USA), beta-Actin-peroxidase (1:5000, #a3854, Sigma-Aldrich), phospho-AKT (Ser505) (1:1000, #4060, Cell Signaling Technology), phospho-AKT (Thr423) (1:1000, #2965, Cell Signaling Technology), total AKT (1:1000, #4691, Cell Signaling Technology), phospho-S6K (Thr398) (1:500, #9209, Cell Signaling Technology), total S6K (1:750, #sc-230, Santa Cruz Biotechnology, Dallas, TX, USA), rabbit IgG-peroxidase (1:2000, #a6154, Sigma-Aldrich). Densitometry and quantification were performed using ImageJ .

**DILP2 Immunofluorescence**

Dissected brains were fixed, stained, and mounted following published protocols with DILP2 antibodies provided by P. Leopold (1:800), rat IgG (#A11077, 1:250, Life Technologies), and phalloidin (#A12379, 1:100, Life Technologies). Approximately 10-20 images per brain were taken with a Zeiss CLS microscope and analyzed using ImageJ to measure total fluorescence of individual IPCs in slices with maximum nuclear area.

**References**

1. Ja WW, Carvalho GB, Mak EM, de la Rosa NN, Fang AY, Liong JC, Brummel T and Benzer S. Prandiology of Drosophila and the CAFE assay. Proceedings of the National Academy of Sciences of the United States of America. 2007; 104(20):8253-8256.

2. Wong R, Piper MD, Wertheim B and Partridge L. Quantification of food intake in Drosophila. PloS one. 2009; 4(6):e6063.

3. Rera M, Clark RI and Walker DW. Intestinal barrier dysfunction links metabolic and inflammatory markers of aging to death in Drosophila. Proceedings of the National Academy of Sciences of the United States of America. 2012; 109(52):21528-21533.

4. Rera M, Bahadorani S, Cho J, Koehler CL, Ulgherait M, Hur JH, Ansari WS, Lo T, Jr., Jones DL and Walker DW. Modulation of longevity and tissue homeostasis by the Drosophila PGC-1 homolog. Cell metabolism. 2011; 14(5):623-634.

5. Bahadorani S, Cho J, Lo T, Contreras H, Lawal HO, Krantz DE, Bradley TJ and Walker DW. Neuronal expression of a single-subunit yeast NADH-ubiquinone oxidoreductase (Ndi1) extends Drosophila lifespan. Aging cell. 2010; 9(2):191-202.

6. Cognigni P, Bailey AP and Miguel-Aliaga I. Enteric neurons and systemic signals couple nutritional and reproductive status with intestinal homeostasis. Cell metabolism. 2011; 13(1):92-104.

7. Schneider CA, Rasband WS and Eliceiri KW. NIH Image to ImageJ: 25 years of image analysis. Nature methods. 2012; 9(7):671-675.

8. Geminard C, Rulifson EJ and Leopold P. Remote control of insulin secretion by fat cells in Drosophila. Cell metabolism. 2009; 10(3):199-207.
