# Supplementary material for: Increased longevity mediated by yeast NDI1 expression in Drosophila intestinal stem and progenitor cells
Source: Aging (Albany NY). 2013 Sep 6;5(9):662–81. doi: 10.18632/aging.100595 (PMC3808699; doi:10.18632/aging.100595)
Supplement: Supplementary file 2 [file aging-05-662-s002.doc]

**
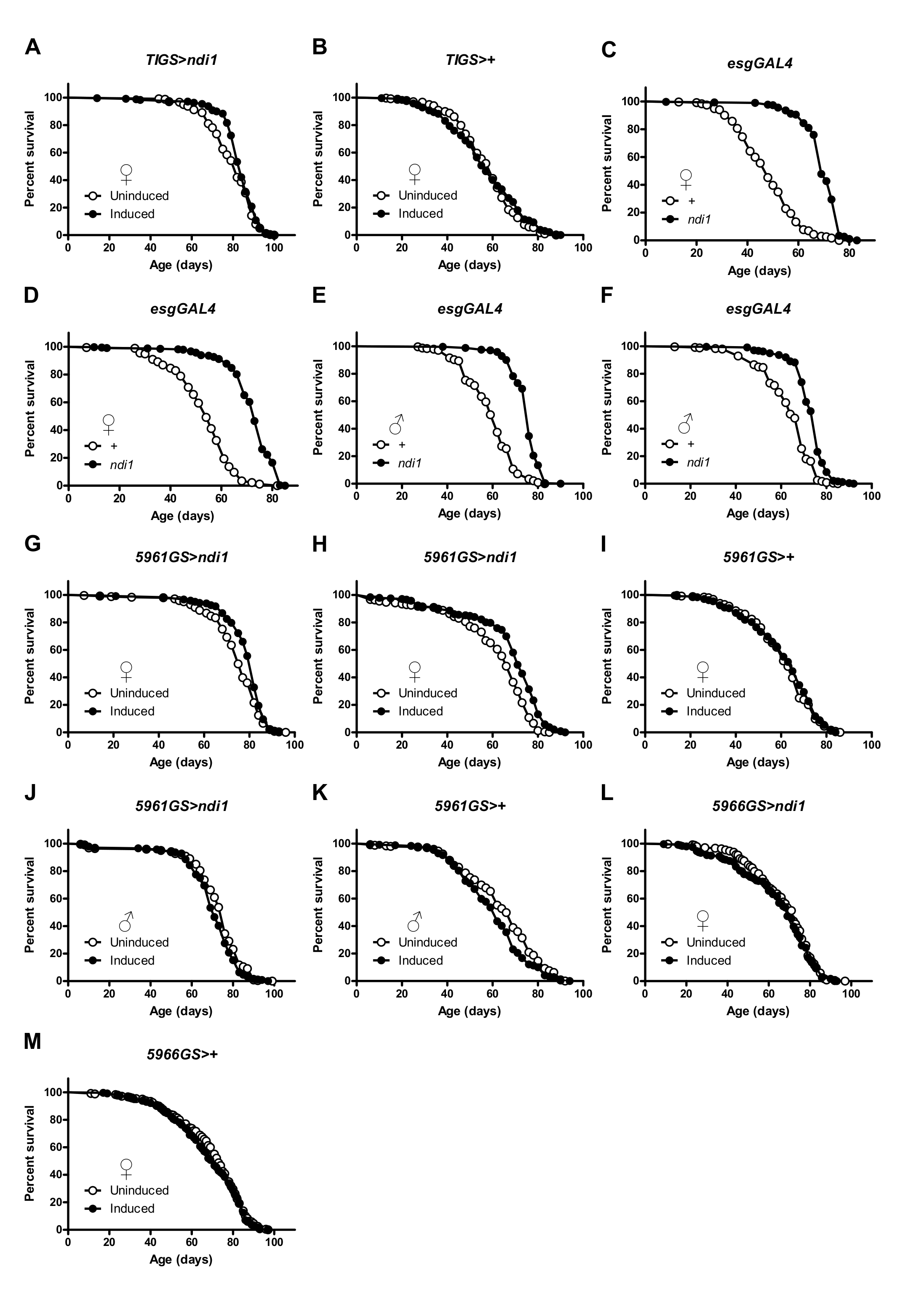
**

**Figure S1. Survival curves of tissue specific *ndi1* expression.**

(A) Replicate survival curve of female *TIGS>ndi1* flies with or without induced transgene expression. Constitutive induction of *ndi1* by RU486 exposure (“Induced”, 10mg/l during development, 50mg/l during adulthood) increases lifespan relative to uninduced controls. (p<0.001, logrank test, 4.2% increase in mean, at least 200 flies per condition).

(B) Survival curve of *TIGS-2* control female flies with or without RU486 exposure. Exposure of *TIGS-2* driver control flies (*TIGS>+*) to RU486 (same conditions as Figure 1B and S1A) does not significantly alter lifespan. (n.s., logrank test, at least 200 flies per condition).

(C-D) Replicate survival curves of female *esgGAL4>ndi1* flies and controls. Expression of *ndi1* in ISCs/EBs (“*ndi1*”, *esgGAL4>ndi1*) increases lifespan relative to controls (“*+*”, *esgGAL4>+*). (p<0.001, logrank test, 46% (C) and 35% (D) increase in mean, at least 170 flies per condition).

(E-F) Survival curves of male *esgGAL4>ndi1* flies and controls. Expression of *ndi1* in ISCs/EBs and testis (“*ndi1*”, *esgGAL4>ndi1*), increases lifespan in mated male flies relative to controls (“*+*”, *esgGAL4>+*). (p<0.001, logrank test, 27% (E) and 16% (F) increase in mean, at least 200 flies per condition).

(G-H) Replicate survival curves of female *5961GS*>*ndi1* flies with or without adult-onset transgene induction. Adult-onset induction of *ndi1* expression in ISCs/EBs by RU486 exposure (0.5mg/l) increases lifespans. (p<0.001, logrank test, 5.6% (G) and 10.5% (H) increase in mean, at least 200 flies per condition).

(I) Survival curve of *5961GS* control female flies with or without adult-onset transgene induction. RU486 (same conditions as Figure S1G and S1H) exposure in *5961GS* control flies (*5961GS>+*) does not alter lifespan. (n.s., logrank test, at least 200 flies per condition).

(J) Survival curve of male *5961GS>ndi1* flies with or without adult-onset transgene induction. Adult-onset induced *ndi1* expression by RU486 exposure (0.5mg/l) results in a mild decrease in lifespan relative to uninduced controls. (p<0.01, logrank test, 2% decrease in mean, at least 200 flies per condition).

(K) Survival curve of *5961GS* control male flies with or without RU486 exposure. Exposure of *5961GS* driver control flies (*5961GS>+*) to RU486 (same conditions as Figure S1J) decreases lifespan. (p<0.01, logrank test, 6% decrease in mean, at least 200 flies per condition).

(L) Survival curve of female *5966GS>ndi1* flies with or without adult-onset transgene induction. Adult-onset induction of *ndi1* expression by RU486 exposure (0.5mg/l) using a geneswitch driver for EBs and ECs, *5966GS*, did not increase lifespan relative to uninduced controls. (n.s., logrank test, at least 200 flies per condition).

(M) Survival curve of *5966GS* control female flies with or without RU486 exposure. Exposure of *5966GS* control flies (*5966GS>+*) to RU486 (same conditions as Figure S1L) does not alter lifespan. (n.s., logrank test, at least 200 flies per condition).

**
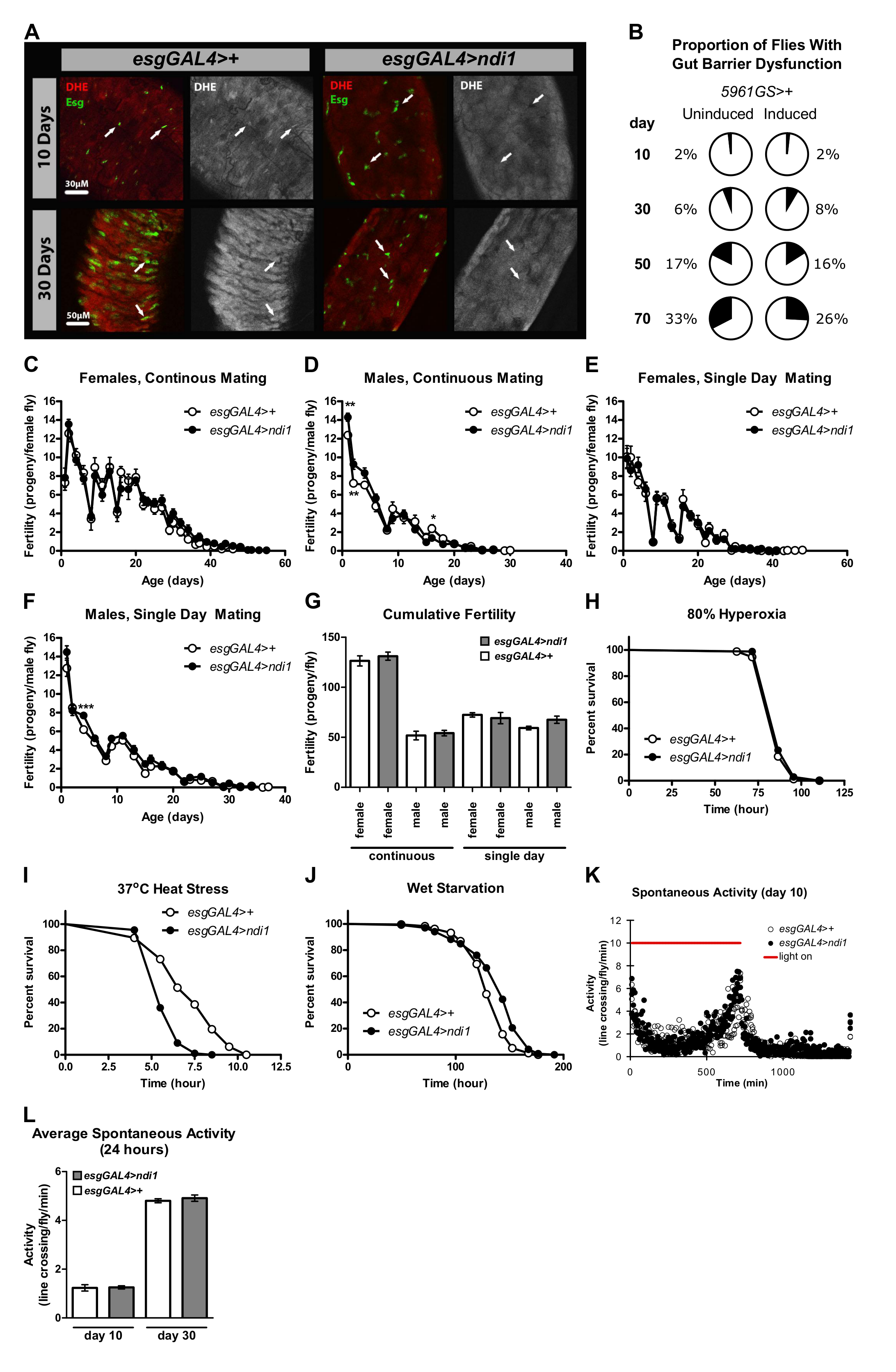
**

**Figure S2. *ndi1* expression does not alter fertility or activity, but affects some stress resistances.**

(A) Single confocal section images of DHE stained midguts evaluating ROS levels during aging. *esgGAL4>ndi1* flies and controls (*esgGAL4>+*), 10 and 30 days post eclosion, were assayed for changes in ROS levels within *esg^+^* cells and per area. (arrows=*esg^+^* cells).

(B) Intestinal integrity in *5961GS* control flies with and without RU486 exposure. Intestinal integrity is not altered by RU486 exposure (same conditions as Figure 2G) in control flies (*5961GS*>*+*). (n.s., binomial test, at least 120 flies per condition).

(C-D) Fertility measurements in continuously mated female (C) and male (D) flies that express *ndi1* in ISCs/EBs. No consistent differences in reproductive output were observed in *esgGAL4>ndi1* flies compared to controls (*esgGAL4>+*). (**p<0.01, *p<0.05, t test, 7-8 replicates per condition, 10 male and 10 female flies per replicate).

(E-F) Fertility measurements in single day mated female (E) and male (F) flies that express *ndi1* in ISCs/EBs. No consistent differences in reproductive output were observed in *esgGAL4>ndi1* flies compared to controls (*esgGAL4>+*). (***p<0.001, t test, 7-8 replicates per condition, 10 female flies per replicate).

(G) Total lifetime reproductive output of flies that express *ndi1* in ISCs/EBs. Expression of ndi1 in ISCs/EBs (“*ndi1*”, *esgGAL4>ndi1*) did not alter total lifetime fertility relative to controls (“*+*”, *esgGAL4>+*). (n.s., t test, 7-8 replicates per condition, as described in Figure S2C-F).

(H) Survival curve of *esgGAL4>ndi1* flies and controls under hyperoxic conditions (80% O­_2_). Flies that express *ndi1* in ISCs/EBs (*esgGAL4>ndi1*) and controls (*esgGAL4>+*) showed similar survival under hyperoxia. (p<0.05, logrank test, 1.6% increase in mean, at least 200 flies per condition).

(I) Survival curve of *esgGAL4>ndi1* flies and controls under heat stress (37°C). Flies that express *ndi1* in ISCs/EBs (*esgGAL4>ndi1*) are more sensitive to heat stress relative to controls (*esgGAL4>+*). (p<0.001, logrank test, 18% decrease in mean, at least 170 flies per condition).

(J) Survival curve of female *esgGAL4>ndi1* flies and controls under wet starvation. Flies that express *ndi1* in ISCs/EBs (*esgGAL4>ndi1*) are more resistant to water-only starvation conditions than controls (*esgGAL4>+*). (p<0.001, logrank test, 6% increase in mean, at least 200 flies per condition).

(K) Spontaneous activity profiles of female *esgGAL4>ndi1* flies and controls. At day 10 of adulthood, activity profiles of flies that express *ndi1* in ISCs/EBs (*esgGAL4>ndi1*) and controls (*esgGAL4>+*) are similar. (3 replicates per condition, approximately 30 flies per replicate).

(L) Average 24-hour spontaneous activities of female *esgGAL4>ndi1* flies and controls. Flies that express *ndi1* in ISCs/EBs (“*ndi1*”, *esgGAL4>ndi1*) and controls (“+”, *esgGAL4>+*) show similar 24-hour spontaneous activities at day 10 and 30 of adulthood. (n.s., t test, 3 replicates per condition, approximately 30 flies per replicate).

**
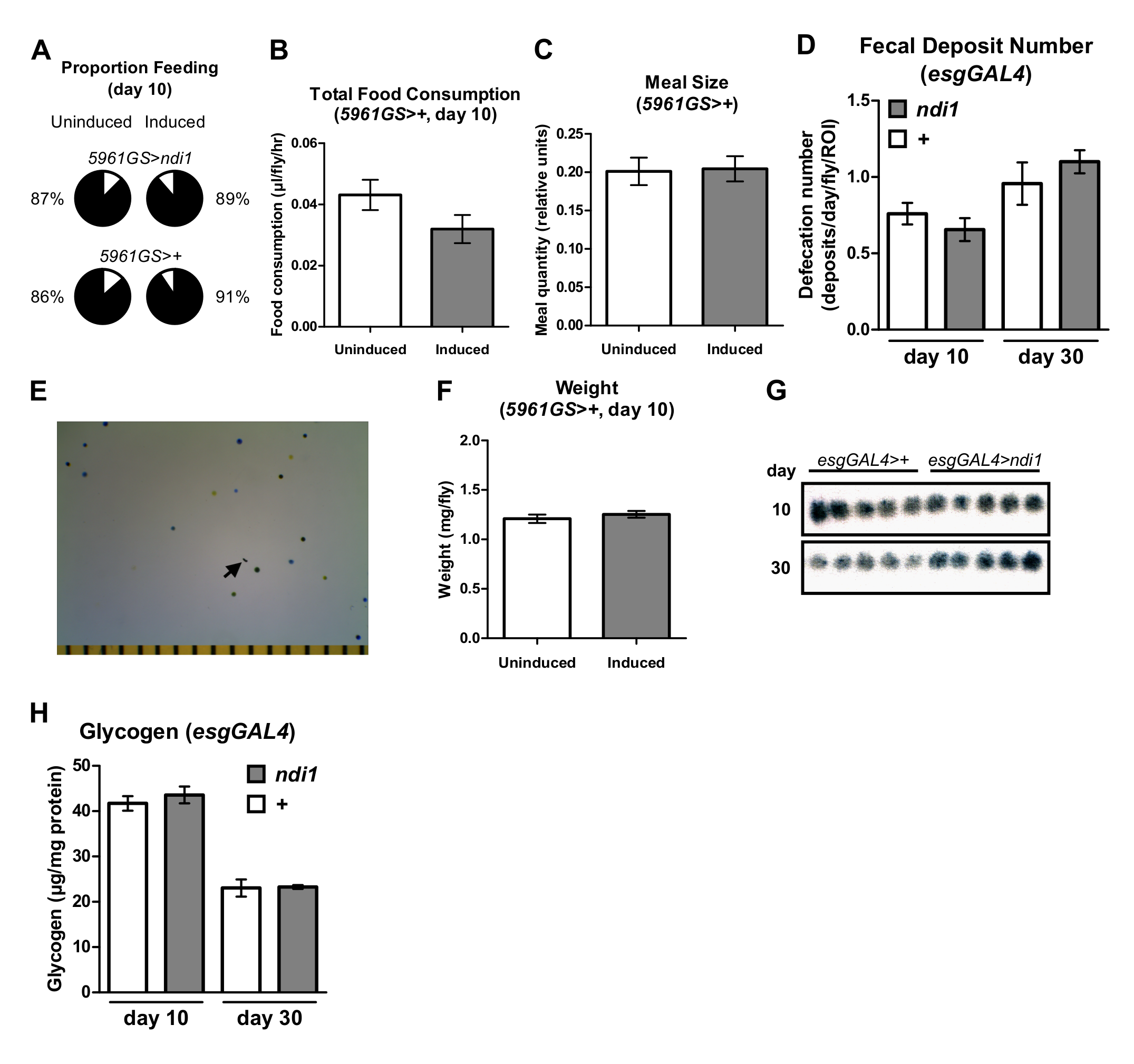
**

**Figure S3. RU486 exposure does not affect feeding or weight in controls, and *ndi1* expression does not alter excreta number or glycogen content.**

(A) Analysis of feeding proportion using a colorimetric dye-tracking assay (adult only *ndi1* expression). At day 10 of adulthood, the proportions of flies that feed are not altered by adult-onset induction of *ndi1* in ISCs/EBs by exposure to RU486 (*5961GS>ndi1*, 0.5mg/l) or by RU486 exposure alone (*5961GS>+*, 0.5mg/l). (n.s., binomial test, 96 flies per condition).

(B) Analysis of total food consumption in driver controls using a CAFE assay. Exposure to RU486 (same conditions as Figure 3D) does not affect total feeding in control flies (*5961GS*>*+*) at day 10 of adulthood. (n.s., t test, 6 replicates per condition, 10 flies per replicate).

(C) Analysis of meal size in control flies using a colorimetric dye-tracking assay. Meal sizes of control flies (*5961GS>+*) are not altered by exposure to RU486. (n.s., t test, approximately 85 flies that ate during the assay period in (A)).

(D) Excreta quantification in *esgGAL4>ndi1* flies and controls. Total number of fecal deposits are similar in flies that express *ndi1* in ICSs/EBs (“*ndi1*”, *esgGAL4>ndi1*) and controls (“*+*”, *esgGAL4>+*). (n.s., t test, 9-10 replicates per condition, 10 flies per replicate).

(E) Representative image of fecal deposits collected from flies cultured on BPB medium. Excreta were used for fecal deposit number (Figure S3D), shape (Figure 3F), and pH analyses (Figure 3G). (Tick marks=1mm, arrow=ROD).

(F) Weights of controls with or without RU486 exposure. Adult-onset exposure to RU486 (same conditions as Figure 3I) does not alter weight in controls (*5961GS>+*) at day 10 of adulthood. (n.s., t test, 6 replicates per condition, 10 flies per replicate).

(G) Image of thin-layer chromatography for triacylglyceride content. Thin-layer chromatography was used to assay triacylglyceride content in flies that express *ndi1* in ISCs/EBs (*esgGAL4>ndi1*) and controls (*esgGAL4>+*) (densitometry in Figure 3K). (5 flies per lane).

(H) Glycogen content as a function of age. Glycogen content is similar in flies that express *ndi1* in ISCs/EBs (“*ndi1*”, *esgGAL4>ndi1*) and controls (“*+*”, *esgGAL4>+*). (n.s., t test, 5 replicates per condition, 5 decapitated flies per replicate).

**
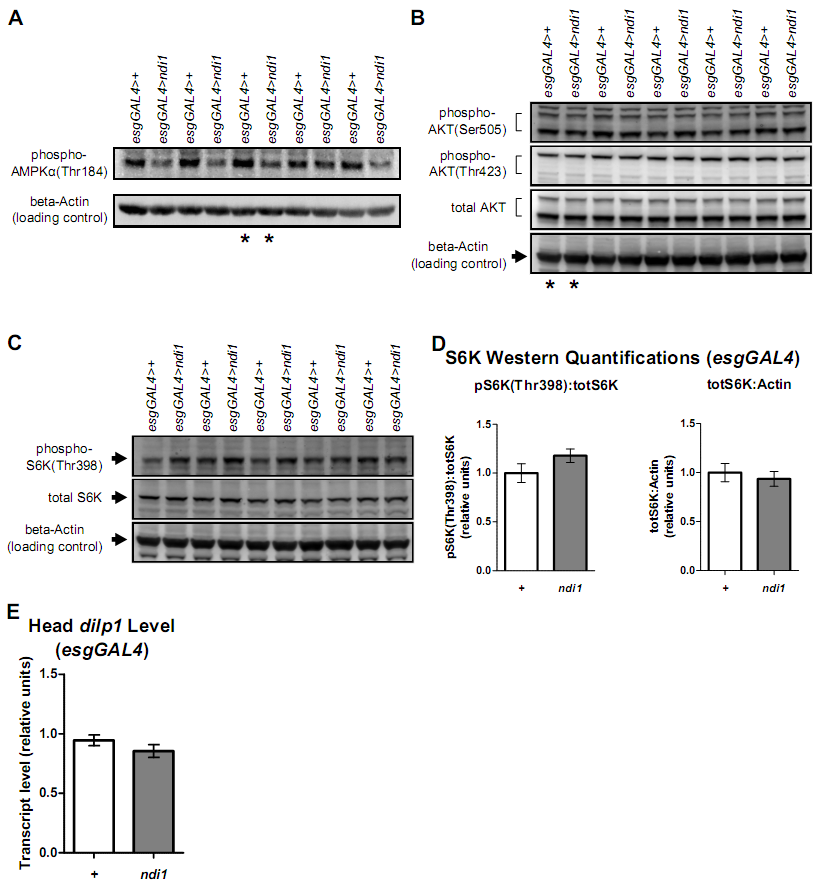
**

**Figure S4. Western blots and head transcript levels of *esgGAL4>ndi1* flies**

(A) Western blot detection of phosphoryated AMPKα(Thr184) and beta-Actin in whole flies. Flies that express *ndi1* in ISCs/EBs (*esgGAL4>ndi1*) and controls (*esgGAL4>+*) were assayed at day 10 of adulthood for phosphorylated AMPKα(Thr184) levels by densitometric analysis (Figure 4B). (*=lanes used for representative image in Figure 4A, 15 flies per lane).

(B) Western blot detection of phosphorylated AKT(Ser505), phosphorylated AKT(Thr423), total AKT, and beta-Actin in whole flies. Flies that express *ndi1* in ISCs/EBs (*esgGAL4>ndi1*) and controls (*esgGAL4>+*) were assayed at day 10 of adulthood for phosphorylated AKT(Ser505), phosphorylated AKT(Thr423), and total AKT levels by densitometric analysis (Figure 4E). (*=lanes used for representative images in Figure 4D, 5 flies per lane).

(C) Western blot detection of phosphorylated S6K(Thr398), total S6K, and beta-Actin in whole flies. Flies that express *ndi1* in ISCs/EBs (*esgGAL4>ndi1*) and controls (*esgGAL4>+*) were assayed at day 10 of adulthood for phosphorylated S6K(Thr398) and total S6K levels by densitometric analysis (Figure S4D). (5 flies per lane).

(D) Densitometric analysis of phosphorylated S6K(Thr398). S6K phosphorylation (normalized to total S6K) is not significantly different at day 10 of adulthood in flies that express *ndi1* in ISCs/EBs (“*ndi1*”, *esgGAL4>ndi1*) relative to controls (“*+*”, *esgGAL4>+*). Total S6K levels (normalized to beta-Actin) are also similar. (n.s., t test, 5 replicates per condition, 5 flies per replicate).

(E) Transcript levels of *dilp1* from heads. Flies that express *ndi1* in ISCs/EBs (“*ndi1*”, *esgGAL4>ndi1*) and controls (“*+*”, *esgGAL4>+*) have similar *dilp1* transcript levels in heads at day 10 of adulthood. (n.s., t test, 5 replicates per condition, 30 heads per replicate).
